# ZnO–Graphene Oxide Nanocomposite for Paclitaxel Delivery and Enhanced Toxicity in Breast Cancer Cells

**DOI:** 10.3390/molecules29163770

**Published:** 2024-08-09

**Authors:** Lorenzo Francesco Madeo, Christine Schirmer, Giuseppe Cirillo, Ayah Nader Asha, Rasha Ghunaim, Samuel Froeschke, Daniel Wolf, Manuela Curcio, Paola Tucci, Francesca Iemma, Bernd Büchner, Silke Hampel, Michael Mertig

**Affiliations:** 1 Kurt-Schwabe-Institut für Mess- und Sensortechnik Meinsberg e.V., 04736 Waldheim, Germany; christine.schirmer@ksi-meinsberg.de (C.S.); michael.mertig@tu-dresden.de (M.M.); 2 Leibniz Institute for Solid State and Material Research Dresden, 01069 Dresden, Germany; s.froeschke@ifw-dresden.de (S.F.); d.wolf@ifw-dresden.de (D.W.); b.buechner@ifw-dresden.de (B.B.); s.hampel@ifw-dresden.de (S.H.); 3 Department of Pharmacy Health and Nutritional Science, University of Calabria, 87036 Rende, Italy; giuseppe.cirillo@unical.it (G.C.); manuela.curcio@unical.it (M.C.); paola.tucci@unical.it (P.T.); francesca.iemma@unical.it (F.I.); 4 Department of Applied Chemistry and Biology, Palestine Polytechnic University, Hebron P.O. Box 198, Palestine; 194591@ppu.edu.ps (A.N.A.); rgonaim@ppu.edu (R.G.); 5 Institute of Solid State and Materials Physics, Technische Universität Dresden, 01062 Dresden, Germany; 6 Institute of Physical Chemistry, Technische Universität Dresden, 01062 Dresden, Germany

**Keywords:** zinc oxide nanoparticles, graphene oxide, nanocomposite, cancer therapy, Paclitaxel delivery

## Abstract

A ZnO-Graphene oxide nanocomposite (Z-G) was prepared in order to exploit the biomedical features of each component in a single anticancer material. This was achieved by means of an environmentally friendly synthesis, taking place at a low temperature and without the involvement of toxic reagents. The product was physicochemically characterized. The ZnO-to-GO ratio was determined through thermogravimetric analysis, while scanning electron microscopy and transmission electron microscopy were used to provide insight into the morphology of the nanocomposite. Using energy-dispersive X-ray spectroscopy, it was possible to confirm that the graphene flakes were homogeneously coated with ZnO. The crystallite size of the ZnO nanoparticles in the new composite was determined using X-ray powder diffraction. The capacity of Z-G to enhance the toxicity of the anticancer drug Paclitaxel towards breast cancer cells was assessed via a cell viability study, showing the remarkable anticancer activity of the obtained system. Such results support the potential use of Z-G as an anticancer agent in combination with a common chemotherapeutic like Paclitaxel, leading to new chemotherapeutic formulations.

## 1. Introduction

Breast cancer is the most commonly diagnosed cancer globally, representing a quarter of all cancer cases and being the second leading cause of cancer death among women [1,2]. The most prevalent therapies for this condition are surgery and chemotherapy, while other alternatives, such as hormone therapy, radiation, and targeted therapy, are also available. Chemotherapy may be administered as a standalone treatment or in conjunction with other therapies, depending upon the specific cancer type and its extent of metastasis. During the first phase of triple-negative breast cancer, chemotherapy might be crucial as a neoadjuvant treatment, preserving or decreasing the size of a tumor prior to surgical extraction. Nevertheless, there have been reports indicating a heightened increase in drug resistance for this kind of breast cancer. Furthermore, the primary drawbacks of conventional chemotherapy are the occurrence of adverse reactions, which intensify as the frequency of medication administration rises, and the frequent reappearance of the disease.

Among the available drugs currently approved for breast cancer chemotherapy, Paclitaxel (Ptx) is one of the most widely used because of its well-recognized activity [3]. Nevertheless, Ptx proved to be a potential burden for patients undergoing chemotherapy, inducing some adverse effects such as peripheral neuropathy, myelosuppression, and hypersensitivity reactions. Additionally, the development of drug resistance to Ptx has also been observed in some cases, further complicating treatment options [4].

The utilization of bioactive and therapeutic agents in cancer therapy could be improved through the implementation of various drug delivery nanomaterials, designed to improve the pharmacokinetic profiles of the agents and suppress their non-target tumor specificity [5]. These systems, comprising organic materials, such as polymer and lipid nanoparticles, as well as inorganic materials, such as metal, carbon, and silica nanoparticles, are organized in various shapes, including spheres, rods, prisms, and sheets [6,7].

GO (graphene oxide) is an oxidized form of graphene obtained via the oxidation of graphite [8]. It possesses some of the key properties of the parent material due to the presence of the hexagonal network of the sp^2^ carbon atom single layer or multiple layers while also being characterized by water dispersibility and superior biocompatibility features due to the presence of a large number of functional oxygen-rich groups like epoxide, hydroxyl (the sheets’ basal planes), carbonyl, and carboxyl (the sheets’ edges) groups [9,10]. These oxygen-rich groups on the surface of GO allow for easy functionalization and tailored modification routes [11,12], making it a versatile material for various applications in different technological fields, including electronics, energy storage, and biomedicine. Additionally, the unique properties of GO, such as its high surface area and excellent mechanical strength, make it an ideal candidate for use in composite materials to enhance their performance and durability. The scientific community widely acknowledges the advantages of using GO as a platform for drug delivery [13,14], with the developed nanocarriers benefiting from the high loading capacity of drug molecules. The basal plane of the GO sheet indeed facilitates π–π interactions, whereas the oxygen-rich functionalities promote electrostatic and hydrogen bonding [15]. The GO-based nanocarriers also take advantage of GO’s good optical (absorption) and photothermal (conversion) properties [16].

At the same time, different metal oxide nanoparticles (e.g., Fe_3_O_4_, NiO, CoO, ZnO, and SnO_2_) have been shown to possess favorable properties for drug delivery due to their intrinsic therapeutic activity (photothermal or photodynamic effects) and ability to be easily incorporated into hydrophobic and hydrophilic carrier systems, as well as their facile derivatization with bioactive molecules [17,18,19]. In particular, ZnO nanoparticles (ZnO NPs) have been widely explored for biomedical applications based on their relatively low toxicity, inherent nutritional benefits, and biodegradability [20,21,22]. Zinc is a micronutrient that is necessary for the activation of many enzymes involved in cellular development, protein and DNA production, and tissue repair. Similarly, ZnO NPs exhibit biocompatibility with human cells owing to their minimal dissolution rate under healthy conditions (pH = 7.4). However, they become toxic when exposed to slightly acidic pH levels since this triggers the fast release of Zn^2+^ ions. This release leads to oxidative stress and cellular damage. Given that the acidity of the extracellular medium is a characteristic of the tumor environment, this particular aspect plays a crucial role in directing toxicity towards cancer cells [23,24]. ZnO NPs have been used as both vehicles for drugs and as an active toxic substance against triple-negative breast cancer (TNBC) cell lines, with their effectiveness being influenced by their form and size.

ZnO NPs have been produced using a range of techniques, including thermal decomposition, sol–gel approaches, hydrothermal procedures, ultrasonication, and green synthesis [25]. The combination of GO with metal oxide nanoparticles led to the development of nanocomposite systems with improved biomedical performance as a result of the intrinsic effects described above [26,27]. Some key examples of these findings include the use of ZnO/GO nanocomposites as pH-responsive devices for delivering doxorubicin [28] and 5-fluorouracil [6] to breast cancer cells. Additionally, a ZnO/GO/TiO_2_ nanosystem has been shown to effectively vectorize quercetin and curcumin [29].

Here, we aim to develop a ZnO/GO nanocomposite material (Z-G) suitable for the vectorization of Ptx to breast cancer cells in order to prove that the enhancement in this drug’s drug efficiency is related to the presence of both GO and ZnO NPs, synergistically promoting cancer cell death and allowing for the use of reduced Ptx dosages. The nanocomposite was synthesized via thermal treatment, without the use of any toxic solvents and at relatively low temperatures, and extensively characterized in terms of its physicochemical and biological properties. Finally, the efficacy of the Ptx-loaded nanosystem was tested using MDA-MB-231 cancer cells and compared to control samples consisting of uncombined GO or ZnO NPs.

## 2. Results and Discussion

### 2.1. Synthesis and Characterization Procedure

In order to avoid the use of toxic solvents and optimize energy costs, a Z-G nanocomposite was obtained using the hydrothermal method at a relatively low temperature and for a shorter time compared to that for other methods available in the literature [30,31].

Preliminary sonication of both GO and ZnO NPs individually was performed to obtain fine dispersions, which were then mixed and heated for several hours to allow them to properly interact with each other, thus forming the composite. The calcination step on the dried product was used as a substitute for conducting heat treatments on the single components; ZnO, indeed, needed to be treated at high temperatures to promote the oxidation of the residual zinc hydroxide to zinc oxide [32]. Moreover, heating above 200 °C causes a partial reduction of GO via the removal of some labile oxygen groups [33]. Since the loading of hydrophobic drugs on graphene-derived materials, as in the case of GO and reduced GO with Ptx, occurs via π-π stacking and hydrophobic interactions [34], a partial reduction of GO is supposed to be useful for a more efficient delivery of Ptx [35,36,37]. Moreover, it can be hypothesized that the oxygen-derived groups stabilized by interaction with Zn are preserved by elimination through thermal treatment.

For a proper evaluation of the effect of ZnO NPs on the nanocomposite’s physical and chemical properties, another nanocomposite (Z-G*) was prepared for comparative purposes by reducing the ZnO-to-GO ratio by half (see the Section 3 for further details). The ultimate aim of this approach is to underline the effect of the incorporation of ZnO NPs into Z-G, since a lower quantity of ZnO NPs (Z-G*) is expected to influence its physicochemical behavior to a lower extent.

**Figure 1:**
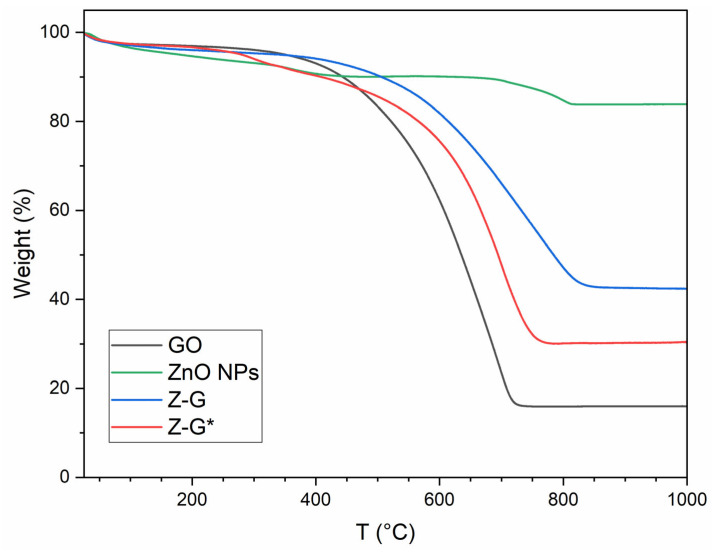
TGA of GO, ZnO NPs, Z-G, and Z-G*.

TGA of GO, ZnO NPs, Z-G, and Z-G* was performed in order to determine the mass content ratios of the individual components in the final composites [22]. By considering the weight percentage loss at 850 °C (the endpoint of the thermal degradation process), the ZnO-to-GO ratio was calculated to be 40:60 for Z-G and 21:79 for Z-G*. A comparison of the experimental and theoretical data showed that a two-fold increase in the ZnO NP content within the reaction feed resulted in an almost proportional increase in the ZnO NPs within the final nanocomposite, highlighting the high affinity of GO for the metal nanoparticles and confirming that the ZnO NP amount was the driving force of the synthetic procedure. Moreover, TGA allowed the thermal stability of the nanocomposite to be evaluated. As shown in Figure 1, all of the analyzed samples showed similar minor weight loss within 100 °C, mainly correlated with the evaporation of adsorbed water.

The ZnO NPs showed a further weight loss between 100 and 400 °C due to the decomposition of Zn(OH)_2_ traces [38]. Z-G and Z-G* composites did not show such weight loss due to the calcination treatment [39], displaying a trend similar to pristine GO. As with pristine GO, they showed a weight loss step at 260 °C due to the decomposition of oxygen functional groups, including carboxyl and hydroxyl groups [40]. In general, the composites showed higher thermal stability compared to GO, and this stability was proportional to the content of ZnO.

**Figure 2:**
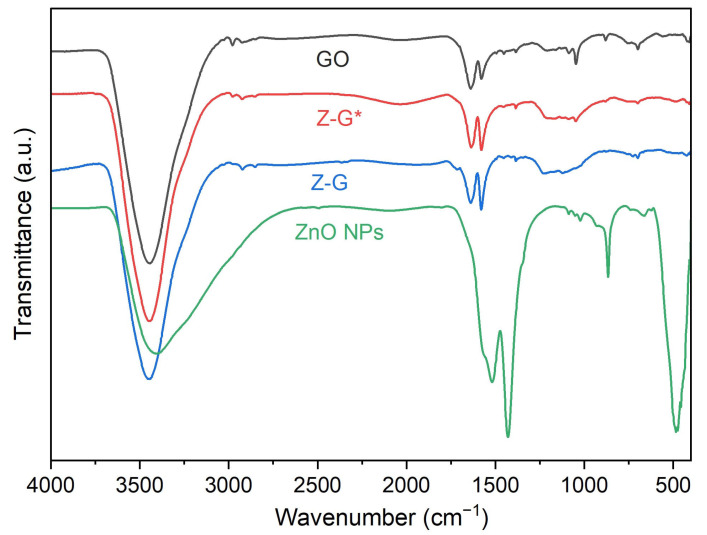
FTIR spectra of GO, ZnO NPs, Z-G and Z-G*.

FTIR spectra (Figure 2) were acquired in the range of 400–4000 cm^−1^ to investigate the vibrational signatures of GO, ZnO NPs, Z-G, and Z-G*.

ZnO NPs showed the absorption peak of the characteristic stretching vibration of the Zn-O bond at 460 cm^−1^. The broad peak at 3000–3300 cm^−1^ is related to the stretching vibrations of O-H residues, thus confirming the presence of unreacted zinc hydroxide in the nanoparticle structures [41], while peaks at 1520 cm^−1^ and 1428 cm^−1^ were attributed to C=O stretching vibrations from unreacted acetate [42].

GO exhibited the O-H vibrational stretching broad peak at 3447 cm^−1^, and further OH-related peaks at 2845 cm^−1^ and 2917 cm^−1^, assigned to the stretching of alcohol and carboxyl groups. The peak at 1625 cm^−1^ was assigned to C=O stretching [43], and that at 1575 cm^−1^ was assigned to the aromatic C=C bonds [44]. At 1385 cm^−1^, 1226 cm^−1^, and 1126 cm^−1^, the O-H bending and C–O stretching vibrations of COOH groups can be observed [43]. The signal at 702 cm^−1^ can be ascribed to C=C vibrational bending.

Both the Z-G and Z-G* composites showed similar spectra compared to GO. Minor shifts to 2975 cm^−1^ and 2973 cm^−1^ were observed for O-H vibrational stretching from alcohol and carboxyl groups. Even if the presence of zinc oxide was later confirmed, only a weak signal characteristic of Zn-O was observed in the 400–600 cm^−1^ region (416 cm^−1^), probably due to the presence of graphene layers over the ZnO NPs [45].

**Figure 3:**
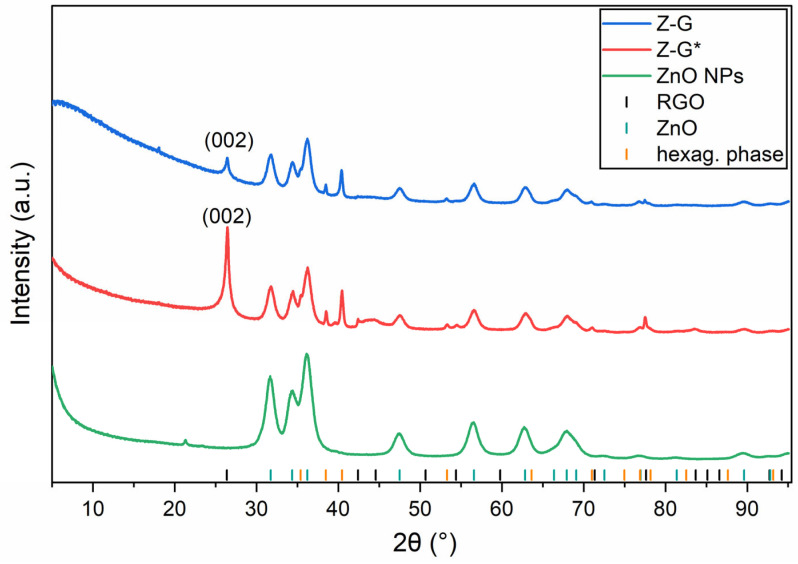
pXRD pattern of ZnO NPs, Z-G, and Z-G*.

In order to investigate the crystal structures of the samples and determine their crystallite sizes, pXRD was performed on the ZnO NPs, Z-G, and Z-G*(Figure 3).

All of the detected reflections of the synthesized ZnO NPs are consistent with the given JCPDS data (36-1451), corresponding to the wurtzite phase. The same reflections were observed in both patterns of Z-G and Z-G*. The intense reflection at 26.38° in the patterns of the composite samples can be ascribed to a reduced GO phase [46,47]. The appearance of several low-intensity reflections similar to those of pure graphite in the same patterns indicate at least a partial graphitic structure, while the d-spacing of the 002 reflex at 26.38° is slightly larger than the value of regular graphite reported in the literature (3.372 Å vs. 3.3555 Å) [48]. This expanded interlayer distance supports the claim of a partial reduction of GO with some functional groups still present that expand the interlayer distance of the stacked 2D layers. Furthermore, the patterns of the composites showed several additional reflections that could not be assigned to regular ZnO, GO, or graphite. However, an investigation of these reflex positions indicated a hexagonal Bravais lattice with lattice parameters that were just 90% of those of the regular ZnO phase, both for a and c. This could be explained by an isotropic partial reduction of regular ZnO during the calcination process. The fact that the diffraction patterns of the composite samples show reflections of regular ZnO and this additional phase suggests that the reduction process might be limited to the surface of the crystallites and caused by a reaction with the reduced GO during the calcination step.

According to the Scherrer equation, the observed reflections of ZnO were especially broadened due to the nanosized crystallites. The equatorial crystallite sizes of the ZnO NPs, Z-G, and Z-G* were determined to be 8.7 ± 0.01, 11.7 ± 0.1, and 12.8 ± 0.2 nm, respectively, whereas the axial sizes were 8.8 ± 0.1, 12.1 ± 0.2, and 13.1 ± 0.3 nm, respectively.

**Figure 4:**
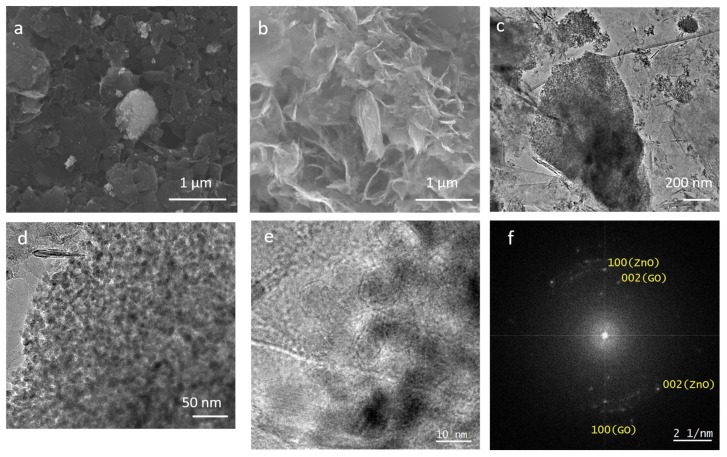
SEM (**a**,**b**) and TEM (**c**–**e**) images of Z-G* (**a**) and Z-G (**b**–**e**). (**c**) Bright-field TEM (BFTEM) image of ZnO NPs clusters on GO sheets. The wrinkling of the latter is visible as dark lines. (**d**) BFTEM images at higher magnification showing morphology, size, and arrangement of the ZnO NPs within the cluster. (**e**) High-resolution TEM (HRTEM) images of ZnO NPs at the edge of the cluster showing lattice planes at a few NPs. (**f**) Fourier transform of (**e**) revealing reflections of both ZnO and GO.

The investigation of Z-G and Z-G* with SEM/EDX allowed us to confirm the presence of the individual components, namely, ZnO and GO, and analyze their morphologies. The Z-G* image (Figure 4a) shows a very high presence of unmodified stacked GO flakes, with some of them partially or fully coated by ZnO NPs. When the ZnO-to-GO ratio increases, as in the Z-G composite (Figure 4b), the morphology of the composite changes, with widespread presence of curls and wrinkles being associated with the partial reduction of GO [49].

TEM allowed for morphological studies and size estimation of the ZnO NPs in the Z-G nanocomposite. In Figure 4c, the ZnO NP agglomerates can be seen on thin, wrinkled graphene oxide flakes. At higher magnification (Figure 4d), the quasi-spherical shape of the ZnO NPs can be observed, with their sizes being determined by random inspection in a range from 5 to 20 nm in diameter. These observations suggest that the particle size corresponds to the crystallite size. We zoomed in on individual ZnO NPs at the edge of the ZnO NP cluster using HRTEM (Figure 4e), and the crystal structure was confirmed by resolving lattice planes at ZnO NPs oriented close to a low-index zone axis. The Fourier transform of the HRTEM image (Figure 4f) reveals reflections corresponding to the 100 and 002 ZnO lattice planes but also reflections stemming from the 100 and 002 GO lattice planes, thus proving the close arrangement of both ZnO NPs and GO flakes.

**Figure 5:**
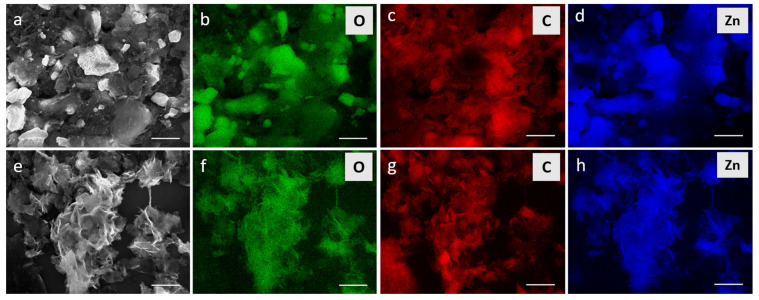
EDX mapping of Z-G* (**a**–**d**) and Z-G (**e**–**h**). The original SEM images are shown in (**a**,**e**). The detected elements were O (**b**,**f**), C (**c**,**g**), and Zn (**d**,**h**). Scale bar = 1 μm.

EDX mapping was used to confirm the presence of the elements in question (O, C, and Zn) and check how they were distributed in the composite. Z-G* mapping images (Figure 5a–d) further confirmed the presence of both coated and uncoated graphene flakes and made the ZnO NP aggregates recognizable, while Z-G mapping (Figure 5e–h) displayed a more homogeneous distribution of the elements.

### 2.2. Ptx Release Studies and Biological Characterization

As already stated, the synthesized Z-G composites were designed as a carrier to target and improve the activity of Ptx and its efficacy as an anticancer agent, with GO and ZnO NPs selected as base materials for the nanoparticle system due to their peculiar properties. GO was expected to extend the release over time and improve carrier-to-drug affinity, while the ZnO NPs were expected to enhance the cytotoxic effect of the drug molecule. Thus, based on the physicochemical characterization showing the higher ZnO NP content in the Z-G nanocomposite compared to the Z-G* nanocomposite, further investigations were performed by using the Z-G composite due to the higher ZnO NP content (see the results of the TGA analyses) and compared to uncombined materials (ZnO NPs and GO).

**Figure 6:**
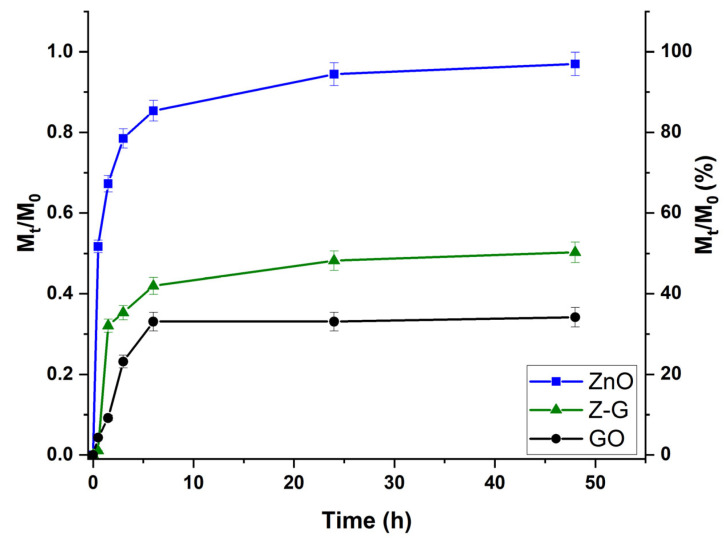
Ptx release profiles for Z-G, GO, and ZnO NP samples.

Before assessing biological efficacy, the Ptx release profiles were determined in a medium mimicking the pH of the tumor micro-environment (Figure 6), with the amount of drug detected in the releasing media expressed according to Equation (1).

(1) $$Ptx\;release=\frac{M_{t}}{M_{0}}$$

where M_t_ and M_0_ are the Ptx amounts (mg) detected at time t and loaded into the Z-G nanocomposite, respectively.

For a more direct comparison of the release profiles from the different materials (Z-G, ZnO NPs, and GO), a fixed Ptx-to-carrier ratio (0.44% by weight) was used in all cases.

The results clearly show that the GO-containing samples possessed higher affinity for the loaded therapeutic, with the amount of Ptx detected in the releasing media not exceeding 50% even after 48 h of incubation, findings that are in agreement with the proposed loading mechanisms, mainly involving π-π stacking and hydrophobic interactions [50]. In greater detail, the maximum release values were found to be 34% and 50% for GO and Z-G, respectively. To better highlight this concept, the experimental data were fitted using an empirical equation proposed in the literature [51], modelling the release of a loaded molecule within a delivery vehicle as a partition between the carrier and the releasing media phases according to reversible first- or second-order kinetics (Equations (2) and (3)):

(2) $$\frac{M_{t}}{M_{0}}=F_{max}(1-e^{-\left(\frac{k_{R}}{F_{max}}\right)t})$$

(3) $$\frac{M_{t}}{M_{0}}=\frac{F_{max}(e^{2\left(\frac{k_{R}}{\alpha }\right)t}-1)}{1-2F_{max}+e^{2\left(\frac{k_{R}}{\alpha }\right)t}}$$

Here, F_max_ represents the maximum amount of Ptx released (M_t_/M_0_), and k_R_ is the release rate constant, while α, a measure of the affinity of the drug towards the solvent, can be calculated using Equation (4):

(4) $$\alpha =\frac{F_{max}}{1-F_{max}}$$

Accordingly, the release rate is a function of the strength of the drug-to-carrier interactions, and drug release can occur only when α > 0.

The kinetic parameters obtained by applying Equations (2) and (3) are reported in Table 1.

It is evident that reversible first-order kinetics are more suitable for modelling the release mechanism (R^2^ > 0.96 for all samples). The higher affinity of the GO-based sample for the cytotoxic drug was confirmed by the significantly lower α values for both GO and Z-G. Moreover, the evaluation of the k_R_ values confirmed that such higher affinity resulted not only in a lower amount of released Ptx (lower F_max_) but also a slower diffusion of the drug molecules from the carrier to the releasing media phases, suggesting potential application for an effective anticancer therapy where sustained release of the drug is preferred [52].

After the determination of the release profiles, with a view to biological characterization, a key aspect to characterize for GO derivatives is their dispersibility in cell culture media. The literature data indeed clearly demonstrate that, although GO and GO derivatives can be well dispersed in an aqueous environment due to the electrostatic repulsion of negatively ionized carboxyl and phenoxyl groups [53], the components of cell culture media can interact with GO, thus modulating its colloidal properties [54]. In particular, fetal bovine serum (FBS), a key component allowing optimal growth and maintenance of cells in in vitro conditions, can bind to the GO surface, leading to the formation of a protein corona [55]. This protein cap gives a new chemical and biological identity to GO, governing its surface properties and thus strongly impacting its biological response [56].

Under the conditions in our experiments, Dynamic Light Scattering (DLS) analyses of a Z-G dispersion (0.5 mg/mL) in cell culture media showed that most of the nanoparticles had a mean hydrodynamic diameter of 396 nm (Figure S1 in the Supplementary Materials), with a minimal subpopulation (3.5%) at 5560 nm, which agrees with the HRTEM observations. Interestingly, no sign of aggregation was recorded over a 72 h period, as reported in previous studies involving GO nanostructures [10]. The presence of a subpopulation within the micrometric range is a well-known intrinsic feature of GO samples [57], and it was confirmed by the DLS measurements of a GO dispersion (0.5 mg/mL) in the cell culture medium (Figure S1 in Supplementary Materials), showing that most of the nanoparticles had a mean diameter of 401 nm and a residual population (7.4%) at 5530 nm. These results are consistent with data from the literature demonstrating that the estimation of GO derivatives’ hydrodynamic diameters via DLS mainly depends on their lateral width [58,59], which was not significantly affected by surface derivatization with the ZnO NPs.

The MDA-MB-231 human breast cancer cell line was selected as an in vitro model for triple-negative, aggressive, hormone-independent breast cancers due to its intrinsic biochemical and metabolic features [60]. Although it is well-known that different cancer cell lines can show different responses to treatment with cytotoxic drugs such as Ptx [61], testing the cytotoxicity on highly malignant MDA-MB-231 cells can give a clear indication of the anticancer activity of a tested nanocarrier material.

MDA-MB-231 cells were treated with GO, Z-G, Ptx, GO/Ptx, ZnO/Ptx, and Z-G/Ptx for 48 h using three different concentrations (a, b, and c) obtained via the dilution of the original dispersion. The nanoparticle concentrations were 12.5 µg/mL, 25 µg/mL, and 50 µg/mL. For each concentration, the loaded nanoparticle dispersion contained 0.055 µg/mL, 0.110 µg/mL, and 0.220 µg/mL of Ptx, respectively.

**Figure 7:**
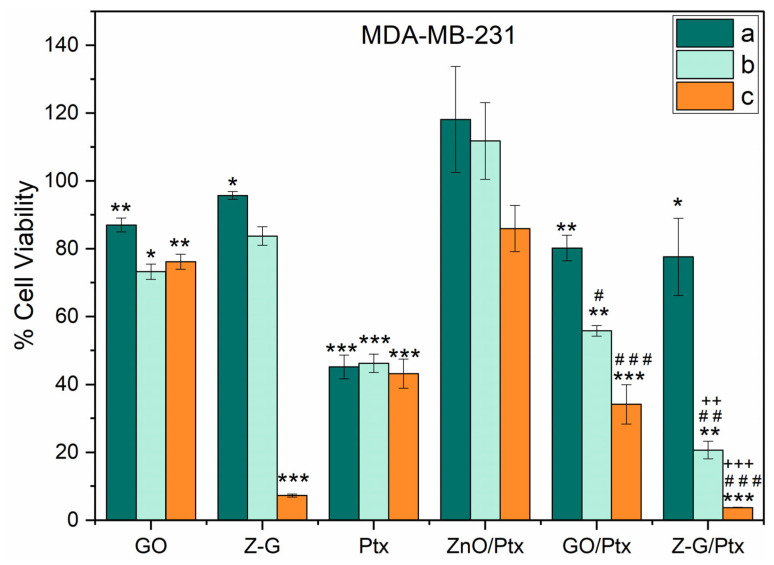
Cancer cells’ viability after 48 h of incubation with unloaded NPs (GO, Z-G), Ptx, and loaded NPs (ZnO/Ptx, GO/Ptx, and Z-G/Ptx) compared to the control (DMSO treatment). ***/**/* decreased viability vs. control (DMSO); ###/##/# decreased viability vs unloaded NPs; +++/++ decreased viability vs Ptx; */# *p* < 0.05; **/##/++ *p* < 0.01; ***/###/+++ *p* < 0.001.

The viability of MDA-MB-231 is reported in Figure 7.

The Z-G carrier was found to be well tolerated at low (a) and intermediate (b) concentrations, while at the highest concentration tested in this study (c), the carrier was toxic; thus, this value cannot be considered when designing a potential Ptx-based anticancer treatment. These results were consistent with those obtained when MCF-10A cells were used as an in vitro model for healthy cells (Figure S2 in the Supplementary Material). Here, negligible cytotoxicity was recorded for samples “a” and “b” (with viability higher that 97%), while sample “c” was found to reduce cell viability by up to 41%, a further confirmation that only samples at low and intermediate Ptx equivalent concentrations should be considered. It should be underlined that, upon treatment with the empty carrier, a more relevant cytotoxic effect was recorded in the MDA-MB-231 cells than in the MCF-10A cells, a finding consistent with data from the literature suggesting that GO-based materials have the ability to hinder the proliferation of cancer stem cells in a wide array of cancers while not being toxic to normal pluripotent stem cells [62,63]. The different metabolic rates of cancer and healthy cells are usually noted as one of the main mechanisms underlying the different responsivities of cell lines to nanomaterials [64].

**Figure 8:**
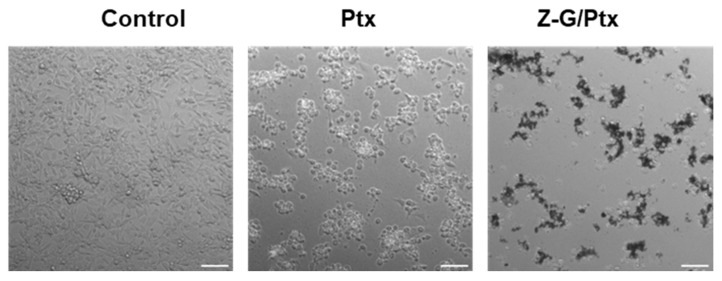
Light microscopy images of cancer cells treated with Ptx (50 μg/mL) and Z-G/Ptx (25 μg/mL) for 48 h. Scale bar = 100 µm.

At the intermediate concentration (b, 0.110 µg/mL), Ptx reduced viability to almost 45.8%, while the Z-G/Ptx samples showed an enhanced toxicity, reducing the number of viable cells to 20.4% with the same amount of loaded Ptx (*p* ˂ 0.01). The ability of the Z-G nanocomposite to enhance the anticancer activity of Ptx, enabling the use of lower concentrations of the drug (with a potential benefit in terms of side effect reduction), was clearly underlined by investigating the cell morphology (Figure 8).

Here, increased signs of a cytotoxic reaction and cell detachment for Z-G/Ptx compared to Ptx alone at this concentration can be detected.

Similar results regarding growth inhibition were reported by other researchers employing ZnO nanoparticles [65] in a comparable Ptx concentration range. Nevertheless, the number of nanoparticles employed was significantly higher, which supports the idea that the use of the proposed nanocomposite is a valuable strategy for reducing the quantity of individual components and their potential toxicities. Furthermore, ZnO and GO showed different results as individual nanocarriers; ZnO/Ptx showed only a minor toxic effect at the highest concentration, reducing the number of viable cells to 86%, while GO/Ptx showed a more pronounced effect, with a clear concentration-dependent activity at the highest to the lowest concentrations used. Such results further hint at a role of GO in interacting with a hydrophobic drug such as Ptx and delivering it to the target cells via π-π interactions.

The obtained data, showing that the Ptx-loaded nanosystem exhibits significantly higher cytotoxicity towards cancer cells compared to the control samples, proved the potential efficiency of the proposed strategy for developing an effective Ptx therapeutic protocol to fight triple-negative breast cancer, leading to increased cancer cell death even at reduced Ptx dosages. Nevertheless, further experiments conducted under more relevant conditions (e.g., 3D cell culture), as well as the determination of the safety profile in non-cancerous cells and in suitable in vivo models, are required before planning a bench-to-clinic translation.

## 3. Materials and Methods

### 3.1. Synthesis of ZnO NPs and Z-G Nanocomposite

ZnO NPs were synthesized according to Meulenkamp with minor modifications [66]. Specifically, Zn(CH_3_COO)_2_ × 2 H_2_O was dissolved in 100 mL of boiling ethanol to obtain a 5 mmol/L solution. Then, the solution was cooled to 0 °C and sonicated using a tip sonicator (Bandelin electronic GmbH, Berlin, Germany) for 45 min (22% amplitude) while adding 100 mL of ethanolic solution of LiOH × H_2_O (7 mmol) dropwise. The temperature was kept constant between 0 °C and 4 °C through the aid of an ice bath. The obtained milky suspension was centrifuged (8000 rpm, 30 min) (Allegra 64 R, Beckman Coulter, Brea, CA, USA) to collect the precipitate, which was washed with ethanol via centrifugation several times until the supernatant appeared to be clear. The obtained ZnO NP powder was stored at room temperature and dispersed in ddH_2_O (0.5 mg/mL) via bath sonication before composite preparation.

For the nanocomposites’ preparation, first, a 1.0 mg/mL GO water dispersion was prepared via tip sonication for 2 h. Then, appropriate amounts of 0.5 mg/mL of ZnO NPs dispersions (2 mL for Z-G and 1 mL for Z-G*) in water were added to 1.0 mL of the GO dispersion. The mixed dispersions were further sonicated for 1 h via tip sonication (22% amplitude) and thermally treated at 100 °C for 6 h under reflux. The product was vacuum-filtered and dried at 60 °C. The collected powder was then subjected to calcination at 320 °C for 2 h.

GO and all chemicals were purchased from Merck/Sigma Aldrich, Darmstadt, Germany.

### 3.2. Characterization Procedure

Thermogravimetric analysis (TGA) was performed using an SDT Q600 (TA Instruments, Hüllhorst, Germany) under a nitrogen atmosphere with the following conditions: 5 mg initial sample weight, 10 mL min^−1^ N_2_ flow, 25–1000 °C temperature range, and 10 °C min^−1^ heating rate.

Fourier transform infrared spectroscopy (FTIR) spectra were recorded using a Vertex80v (Bruker Optic GmbH, Karlsruhe, Germany) with KBr pellet technique, with 250 scans performed for each spectrum.

Powder X-ray diffraction (pXRD) was performed with Cu–K_α1_ radiation (λ_Kα1_ = 1.54060 Å) in transmission geometry using a STOE STADI-P Ge (111) primary-beam monochromator (STOE & Cie GmbH, Darmstadt, Germany) equipped with a Mythen 1K detector, with a 0.015° step size and a camera distance of 191 mm (Dectris AG, Baden-Daettwil, Switzerland). The crystallite domain sizes of the ZnO NPs were calculated from the diffraction pattern using GSASII version 5311, using a uniaxial domain size model with 001 as the unique axis [67].

Scanning electron microscopy (SEM) images and energy-dispersive X-ray spectroscopy (EDX) measurements of samples were taken with an FEI NOVA NanoSEM 200 (5–15 kV) (Thermo Fisher Scientific, Hillsboro, OR, USA) via deposition of previously water-dispersed samples onto Si/SiO_2_ substrates.

EDX measurements were taken with a “QUANTA 200/400” (AMATEX, FEI, Hillsboro, OR, USA) at 15 kV. For mapping, the images were recorded by performing a full background fitting and peak deconvolution according to the detected elements to ensure a correct intensity assignment for the overlapping peaks at low energies.

High-resolution transmission electron microscopy (HRTEM) was performed using a double-corrected Titan^3^ 80–300 TEM instrument (Thermo Fisher Scientific, USA) operated at 300 kV electron acceleration voltage. Images were recorded on a 4 k by 4 k pixel CMOS camera (Oneview, Gatan-Ametek, Pleasanton, CA, USA).

DLS measurements were taken using a Zetasizer Nano ZS (Malvern Panalytical, Malvern, UK).

### 3.3. Ptx Loading Procedure

Ptx was loaded on Z-G, GO, and ZNO NPs at a fixed Ptx-to-nanocomposite ratio of 0.44% (*w*/*w*) by dispersing, in separate experiments, 5 mg/mL nanocarrier in a Ptx water solution (22 µg/mL, 10% DMSO), followed by 48 h of soaking.

### 3.4. Ptx Release Studies

Release experiments were carried out using a dialysis method, as follows: 1 mL nanoparticle suspensions in ddH_2_O were loaded in a dialysis bag (Spectra/Por, MWCO 3.5 kDa, Fisher Scientific, Milan, Italy) and dialyzed against 14 mL of an acetate buffer (0.01 M, pH 5.5)/ethanol mixture (90/10 by vol) at 37 °C in a beaker with constant stirring [68]. At pre-established times, samples (1 mL) of release medium were withdrawn and replaced with fresh medium. Withdrawn samples were centrifuged (8000 rpm, 30 min) (Allegra 64 R, Beckman Coulter, Brea, CA, USA), and the amount of Ptx in the supernatant was determined via UV-Vis spectroscopy using an Evolution 201 spectrophotometer (ThermoFisher Scientific, Hillsboro, OR, USA) operating with 1.0 cm quartz cells set at 240 nm using a standard calibration curve of Ptx in the same media [69].

The same procedure was performed when ZnO or GO were used as carrier systems.

### 3.5. Cell Culture

MDA-MB-231 cells were used as a model cell line for triple-negative human breast cancer. They were purchased from the DSMZ-German Collection of Microorganisms and Cell Cultures GmbH (Braunschweig, Germany). All MCF-10A human mammary epithelial cells were purchased from American Type Culture Collection (ATCC, Manassas, VA, USA).

MDA-MB-231 culturing was carried out at 37 °C in a humidified atmosphere of 5% CO_2_ in DMEM supplemented with 10% fetal bovine serum, 100 U/mL of penicillin, 100 µg/mL of streptomycin, and 0.25 of µg/mL amphotericin B (all purchased from PAN-Biotech GmbH, Aidenbach, Germany).

MCF-10A cells were cultured in DMEM-F12 supplemented with 10% horse serum, 2 mM of L-glutamine, 1% penicillin/streptomycin, 0.5 mg/mL of hydrocortisone, 20 ng/mL of human epidermal growth factor, and 0.1 mg/mL of cholera enterotoxin (all purchased from Merck/Sigma Aldrich, Darmstadt, Germany).

Cell cultures were tested for the appearance of mycoplasma with MycoStrip Mycoplasma detection kit (InvivoGen, Toulouse, France). One day prior to performing experiments, MDA-MB-231 were seeded in 96-well-plates at 4 × 10^5^ cells/well. Assays were performed at 70–80% confluence.

An Axio observer-Z1 microscope (Carl Zeiss Microimaging, Jena, Germany) equipped with an Evolve-EM 512 camera (Photometrics, Tucson, AZ, USA) was used to take microscopy images of the cells.

### 3.6. Cell Viability Assay

For cells treatments, Ptx-loaded nanocomposite was diluted to obtain three different Z-G concentrations: Z-G/Ptx a, Z-G/Ptx b, and Z-G/Ptx c (with a Ptx-to-carrier ratio of 0.44% by weight in all cases). For comparison, the same procedure was applied for GO (GO/Ptx) and ZnO NPs (ZnO/Ptx) (Table 2).

After 48 h of treatment, cell viability was assessed by colorimetrically measuring mitochondrial activity, as stated in [70]. After being washed, half of the exposed cells were supplied with culture medium, while the other half was killed with medium containing 0.2% Triton X-100 (Dow, Midland, MI, USA). The latter wells were used to eliminate possible interferences from nanoparticles with absorbance readings and/or 4-[3-(4-Iodophenyl)-2-(4-nitro-phenyl)-2H-5-tetrazolio]-1,3-benzene sulfonate (WST-1) substrate since they contained the same amount of test materials but no viable cells. After 15 min of incubation, WST-1 (Roche Diagnostics GmbH, Mannheim, Germany) was added to all wells for 3 h. Absorbance (abs) was measured at 480 nm and 630 nm using a microplate reader BioTek Synergy H1 (Agilent, Santa Clara, CA, USA), and relative cell survival with respect to the control (solvent) was calculated using Equation (5):

(5) $$\frac{{Abs}_{sample}}{{Abs}_{control}}\times 100$$

Unless otherwise stated, all chemicals were purchased from Merck/Sigma Aldrich, Darmstadt, Germany.

### 3.7. Statistical Analysis

Data are presented as means of three replicates with error bars showing standard deviations. For viability assays, to test for the differences in treatments, a two-tailed *t*-test was applied. Values of *p* less than 0.05 were considered statistically significant.

## 4. Conclusions

In this work, we provided experimental proof that a ZnO/GO nanocomposite prepared via thermal treatment was suitable for the vectoring of Ptx, resulting in improved therapeutic effectiveness, as reported in viability assays conducted on MDA-MB-231 breast cancer cells. Z-G was produced using a simple, environmentally friendly synthesis method that did not involve using toxic chemicals or high temperatures. To confirm the stability and content of the produced material, a thorough physico-chemical characterization was carried out using a variety of methods. TGA determined the actual mass content ratio of the composite for each component, and pXRD verified the crystalline nature of the material. Zn, C, and O were uniformly distributed throughout the Z-G nanocomposite according to the results of elemental SEM mapping, while HRTEM and SEM allowed the visualization of the morphology and size of ZnO NPs grafted on graphene flakes in the final material. Biological characterization via viability assays validated the potential role of Z-G as an anticancer agent in synergy with a conventional drug such as Ptx, as suggested by the significant improvement achieved in the treatment of breast cancer cells.

## Figures and Tables

**Table 1:** Fitting parameters for Ptx release from ZnO, GO, and Z-G samples.

Sample	Reversible First-Order Kinetics				Reversible Second-Order Kinetics
	R^2^	F_max_	α	k_R_	R^2^
ZnO	0.9592	0.98	15.6	1.40	0.7958
GO	0.9736	0.35	0.5	0.91	0.8951
Z-G	0.9633	0.48	0.9	0.98	0.9014

**Table 2:** Composition of Ptx-loaded samples.

	a	b	c
[Ptx]	0.055 µg/mL	0.110 µg/mL	0.220 µg/mL
[NPs] ^1^	12.5 µg/mL	25 µg/mL	50 µg/mL

^1^: Z-G, GO or ZnO.

## Data Availability

Data are available within this paper.

## Supplementary Materials

The following supporting information can be downloaded at https://www.mdpi.com/article/10.3390/molecules29163770/s1, Figure S1: Dynamic light scattering (DLS) data on the distribution of the relative intensity of scattered light by particle size (Stokes diameter) for GO and Z-G nanocomposite at concentration 0.5 mg/mL in cell culture media at 25 °C. Figure S2: MCF-10A cells viability after 48 h incubation with unloaded Z-G over control (DMSO treatment). *** *p* < 0.001 vs control.
