# Supplementary material for: ZnO–Graphene Oxide Nanocomposite for Paclitaxel Delivery and Enhanced Toxicity in Breast Cancer Cells
Source: Molecules. 2024 Aug 9;29(16):3770. doi: 10.3390/molecules29163770 (PMC11357239; doi:10.3390/molecules29163770)
Supplement: Supplementary file 1 [file molecules-29-03770-s001.zip › molecules-3088184-supplementary.pdf]

molecules

ZnO–Graphene Oxide Nanocomposite for Paclitaxel Delivery and Enhanced Toxicity in Breast Cancer Cells

Lorenzo Francesco Madeo, Christine Schirmer, Giuseppe Cirillo, Ayah Nader Asha, Rasha Ghunaim, Samuel Froeschke, Daniel Wolf, Manuela Curcio, Paola Tucci, Francesca Iemma, Bernd Büchner, Silke Hampel and Michael Mertig

Supplementary Materials

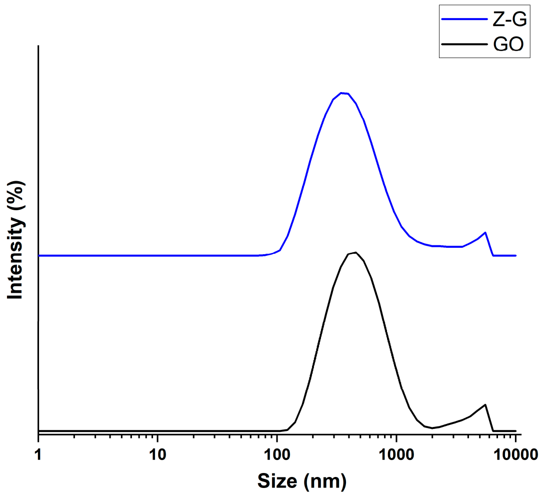

Figure S1. Dynamic light scattering (DLS) data on the distribution of the relative intensity of scattered light by particle size (Stokes diameter) for GO and Z-G nanocomposite at concentration 0.5 mg/mL in cell culture media at 25 °C.

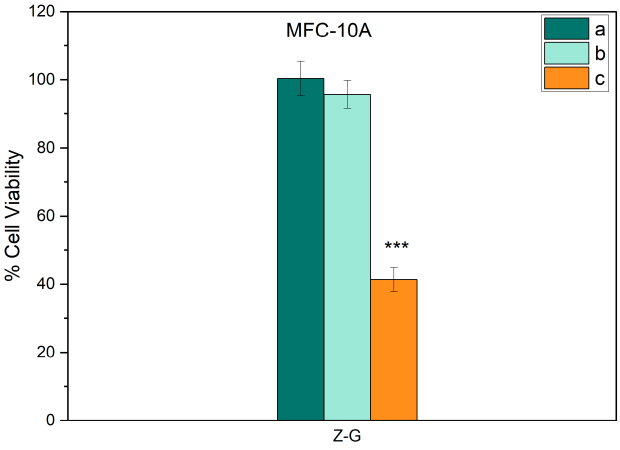

Figure S2. MCF-10A cells viability after 48 h incubation with unloaded Z-G over control (DMSO treatment). *** p < 0.001 *vs* control
